# Amino Acid Residues Contributing to Function of the Heteromeric Insect Olfactory Receptor Complex

**DOI:** 10.1371/journal.pone.0032372

**Published:** 2012-03-05

**Authors:** Tatsuro Nakagawa, Maurizio Pellegrino, Koji Sato, Leslie B. Vosshall, Kazushige Touhara

**Affiliations:** 1 Department of Applied Biological Chemistry, Graduate School of Agricultural and Life Sciences, The University of Tokyo, Tokyo, Japan; 2 Laboratory of Neurogenetics and Behavior, The Rockefeller University, New York, New York, United States of America; 3 Howard Hughes Medical Institute, The Rockefeller University, New York, New York, United States of America; Natural Resources Canada, Canada

## Abstract

Olfactory receptors (Ors) convert chemical signals—the binding of odors and pheromones—to electrical signals through the depolarization of olfactory sensory neurons. Vertebrates Ors are G-protein-coupled receptors, stimulated by odors to produce intracellular second messengers that gate ion channels. Insect Ors are a heteromultimeric complex of unknown stoichiometry of two seven transmembrane domain proteins with no sequence similarity to and the opposite membrane topology of G-protein-coupled receptors. The functional insect Or comprises an odor- or pheromone-specific Or subunit and the Orco co-receptor, which is highly conserved in all insect species. The insect Or-Orco complex has been proposed to function as a novel type of ligand-gated nonselective cation channel possibly modulated by G-proteins. However, the Or-Orco proteins lack homology to any known family of ion channel and lack known functional domains. Therefore, the mechanisms by which odors activate the Or-Orco complex and how ions permeate this complex remain unknown. To begin to address the relationship between Or-Orco structure and function, we performed site-directed mutagenesis of all 83 conserved Glu, Asp, or Tyr residues in the silkmoth BmOr-1-Orco pheromone receptor complex and measured functional properties of mutant channels expressed in *Xenopus* oocytes. 13 of 83 mutations in BmOr-1 and BmOrco altered the reversal potential and rectification index of the BmOr-1-Orco complex. Three of the 13 amino acids (D299 and E356 in BmOr-1 and Y464 in BmOrco) altered both current-voltage relationships and K^+^ selectivity. We introduced the homologous Orco Y464 residue into *Drosophila* Orco *in vivo*, and observed variable effects on spontaneous and evoked action potentials in olfactory neurons that depended on the particular Or-Orco complex examined. Our results provide evidence that a subset of conserved Glu, Asp and Tyr residues in both subunits are essential for channel activity of the heteromeric insect Or-Orco complex.

## Introduction

The detection of odorants and pheromones is essential for insects to find food, avoid predators and noxious agents in the environment, and find appropriate mating partners. Insects sense odorants and pheromones via specialized olfactory sensory neurons (OSNs) located on two sensory appendages on the head, the antennae and maxillary palps. Both of these appendages are covered with specialized sensory hairs called sensilla that house one to four OSNs in the vinegar fly *Drosophila melanogaster* and the silkmoth *Bombyx mori*. The dendritic knobs of OSNs are enriched in membrane-bound odorant receptors (Ors) that play a primary role in recognizing an odorant or a pheromone [1], [2], [3], [4]. The Or expressed in an OSN determines the sensitivity and specificity of the OSN [5], which in turn governs innate and learned olfactory behaviors, such as attraction to food and pheromones and avoidance of repellents [6].

Insects possess 60–400 members of the Or family, which can be divided into three distinct functional classes. The first two classes of Ors are ligand-selective—those that respond to general odorants and a smaller number specialized to detect pheromones [7], [8], [9], [10], [11]. General odorant-selective Ors have little homology between insect species, whereas pheromone receptors in the moth show some homology [9], [12], [13], [14], [15], [16], [17], [18] (**Figure S1A**). The third functional class is a single member of the Or family called Orco, which is highly conserved in all known insect species and functions as an obligate chaperoning co-receptor in complex with ligand-selective Ors [19], [20], [21], [22]. Most OSNs co-express one of the canonical Ors and the Orco family, and these two types of receptors comprise a heteromultimeric complex of unknown stoichiometry [12], [22], [23]

The role of Ors in all animals is to convert chemical signals to electric signals. In vertebrates, Ors are G-protein-coupled receptors (GPCRs), and the odorant ligand activates a signaling pathway that leads to the production of intracellular second messengers and subsequent opening of ion channels [24]. In contrast, how the insect Or-Orco complex converts odorant or pheromone binding to OSN depolarization is less well understood. While insect Ors were initially assumed to be seven transmembrane domain GPCRs, further analysis showed that they lack sequence similarity with known GPCRs [25]. In addition, the membrane topology of insect Ors is inverse to that of GPCRs, with the amino terminus located intracellular and an extracellular carboxy terminus [22], [26] Despite the lack of homology and inverted topology relative to GPCRs, several groups have provided evidence that insect Ors signal through or are modulated by G-proteins [27], [28], [29], [30]. Other studies have reported that G-proteins and cyclic nucleotides are not involved in odor responses *in vivo* or *in vitro* [31], [32], [33]. Therefore, the question of whether insect Ors function like GPCRs or are modulated by G proteins remains controversial. Several groups have recently proposed that the insect Or-Orco complex functions as a novel type of ligand-gated nonselective cation channel [29], [32], [33], [34], [35], [36], which may rely on Gα_s_ and Gα_q_ pathways for function [29], [30].

While there is agreement among these groups that the Or-Orco complex can form a non-selective cation channel, there is not yet a clear consensus on how these membrane proteins function. Wicher et al. proposed that Orco can function as a cyclic nucleotide activated cation channel in the absence of a ligand-selective Or subunit [29]. Jones et al. provided support for this model in their discovery of VUAA1, an allosteric agonist that can activate Orco expressed in heterologous cells without an partner Or subunit [33]. Two other groups demonstrated that the sensitivity of the Or-Orco complex to the cation channel blocker ruthenium red depended on the Or-Orco subunit composition [36]. This suggested that both Or and Orco subunits may contribute to ion permeability of this membrane protein complex. Despite much recent interest in studying the structure and function of insect odorant receptors, a large number of important questions remain unsolved. How do odorants and pheromones activate the Or-Orco complex and how do ions permeate this protein complex? In the heteromeric complex, do both Or and Orco subunits contribute to ion permeability? If so, is it possible to narrow down the regions that contribute to ion permeability? Do such regions contain residues previously associated with ion-conducting pores in ion channels, or does this class of receptors have a completely novel structure?

In the present study, we began to answer these questions by carrying out a comprehensive mutational analysis of all conserved Glu, Asp, and Tyr residues in the Or-Orco complex using the silkmoth bombykol pheromone receptor complex as a model. Our aim was to identify regions in Or and/or Orco that are required for ion permeability and other biophysical properties of this protein complex. Of the 83 conserved residues in Or and Orco mutated, three altered both current-voltage relationships and ion selectivity of the Or-Orco complex. Our results suggest that both the ligand-selective Or subunit and the Orco co-receptor contribute to cation channel activity and that some amino acid residues near the carboxy terminus of both subunits are important for Or-Orco channel function.

## Results

### Site-directed mutagenesis of conserved residues in the insect Or-Orco complex

**Figure 1:**
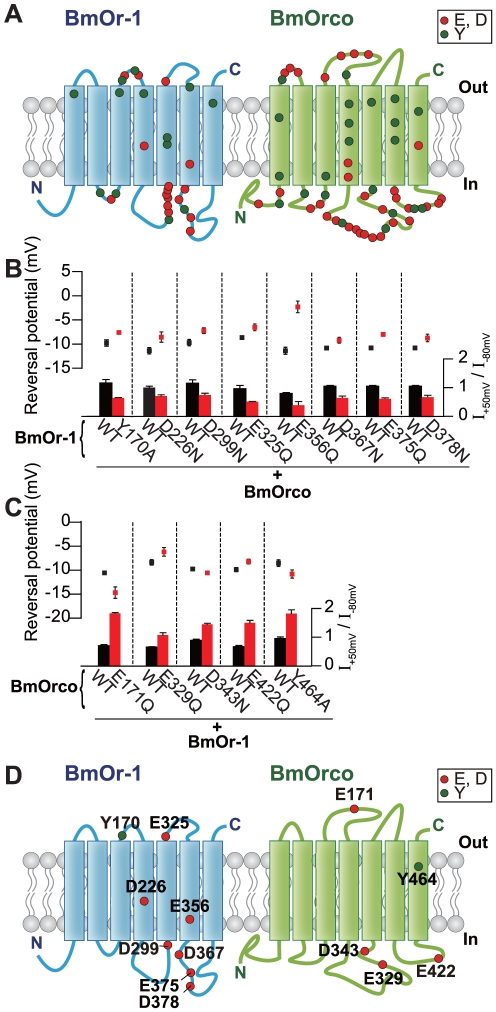
Mutations in BmOr-1 and BmOrco that affected reversal potential and rectification index. (**A**) Schematic of the location of amino acids in BmOr-1 and BmOrco that were mutated in this study. Transmembrane domains were predicted using the PHDhtm algorithm [48]. (**B,C**) Reversal potential (top) and rectification index (bottom) of oocytes expressing mutant BmOr-1 with wild type (WT) BmOrco (B) or WT BmOr-1 with mutant BmOrco (C). Black and red bars and symbols represent WT and mutants, respectively. Only mutants with a significant effect on both reversal potential and rectification index are depicted (unpaired Student's t-test, mutant vs. WT p<0.05). Data on remaining mutants can be found in **Figure S2B**. Data are shown as mean ± S.E.M., n = 8–10. Bombykol was applied at the concentration of 1 µM to each oocyte. (**D**) Schematic showing the eight mutations in BmOr-1 and five mutations in BmOrco that affected both rectification index and reversal potential **(see also Figure S2B,S3A)**.

We carried out a comprehensive mutational screen to identify amino acids in Ors and Orco that are important for odor-evoked function. Many cation channels have Glu, Asp, or Tyr residues in their ion selectivity filters, and these residues play important roles in selective ion permeation [37]. Because the insect Or-Orco complex also forms non-selective cation channels [29], [32], [33], [34], we reasoned that among the conserved Glu, Asp, or Tyr residues, some may be essential for Or-Orco cation channel function. To identify such conserved residues, we first compared the amino acid sequence of the bombykol receptor, BmOr-1 (*Bombyx mori* olfactory receptor 1) [38], with pheromone receptors in other insects (**Figure S1A**). We also compared BmOrco (*Bombyx mori* Orco) with Orco in other insect species (**Figure S1B**). 29 Glu, Asp, or Tyr residues in BmOr-1 and 54 Glu, Asp, or Tyr residues in BmOrco are highly conserved (**Figure 1A**). We used site-directed mutagenesis to mutate all 83 Glu, Asp, and Tyr residues to Gln, Asn and Ala, respectively. Each mutant BmOr-1 or BmOrco contained a single amino acid mutation for a total of 83 individual mutants analyzed in this study. We expressed either mutant BmOr-1 with wild type BmOrco or wild type BmOr-1 with mutant BmOrco in *Xenopus* oocytes and measured current-voltage relationships of responses elicited by bombykol. We quantified a rectification index, defined as the ratio of current amplitude at +50 mV versus −80 mV, and the reversal potential of each mutant Or-Orco combination and compared it to the same parameters in wild type Or-Orco (**Figure 1**). To eliminate confounding effects of biological variation found in oocytes derived from different animals, responses of a given Or-Orco mutant combination were recorded from oocytes derived from the same individual.

### Effects of Glu, Asp, or Tyr mutations in BmOr-1 on ion channel function

Among 29 BmOr-1 mutants tested with wild type BmOrco, the rectification index of 14 (Y109A, Y167A, Y170A, D226N, E274Q, D295N, D299N, Y312A, E325Q, E356Q, D363N, D367N, E375Q, D378N) was decreased compared with wild type, indicating that inward rectification was increased by the mutations (**Figure 1B**, **Figure S2B**). Of these 14 mutants, eight also had reversal potentials that were positively shifted compared to wild type (Y170A, D226N, D299N, E325Q, E356Q, D367N, E375Q, D378N) (**Figure 1B** **, Figure S2B**).

We next asked whether mutations of the corresponding residues in BmOr-3, a *Bombyx mori* bombykal receptor [12], would cause similar changes in ion channel function. We generated and analyzed seven homologous mutations in BmOr-3 (the corresponding residues in BmOr-1>BmOr-3 are: Y170>Y168, D226>D223, D299>D308, E325>E334, E356>E365, D367>D376, E375>E384, and D378>D387) (**Figure S1A**). Oocytes injected with BmOr-3 D308N and wild type BmOrco did not show a response to bombykal, precluding further functional analysis (**Figure S3**). The BmOr-3 E365Q mutation decreased the rectification index and positively-shifted the reversal potential (**Figure S3**), similar to that found for BmOr-1 E356Q (**Figure 1**). The remaining five BmOr-3 mutants except for Y168A tested in combination with wild type BmOrco were impaired in rectification index and reversal potential (**Figure S3**). Based on these results, eight BmOr-1 mutants with altered rectification indices and reversal potentials were further analyzed (Y170, D226, D299, E325, E356, D367, E375, D378) (**Figure 1D**).

### Effects of Glu, Asp or Tyr mutation in BmOrco on ion channel function

Among 54 BmOrco mutants tested in combination with wild type BmOr-1, the rectification index of 17 was different from wild type, with 12 showing increased rectification indices (E120Q, E171Q, D218N, E230Q, E244Q, E269Q, E329Q, D343N, Y413A, Y418A, E422Q, Y464A), and five showing decreased rectification indices (Y103A, E175Q, Y252A, E258Q, D276N) (**Figure 1C**, **Figure S2A–B**). Of these 17 mutants, five (E171Q, E329Q, D343N, E422Q, Y464A) showed positive or negative shifts in reversal potential compared to wild type (**Figure 1C**, **Figure S2A–B**). These five BmOrco mutants were selected for further analysis (**Figure 1D**).

### Effects of Glu, Asp or Tyr mutation in BmOr-1 and BmOrco on ion selectivity

The alteration of amino acids involved in pore formation can alter ion channel selectivity. We therefore performed ion substitution experiments to ask if any of the eight BmOr-1 and five BmOrco mutations that affected the rectification index and reversal potential also affected ion permeability. To calculate permeability ratios, the reversal potential of oocytes expressing wild type and mutants was measured in Na^+^ and K^+^ extracellular solution. We tested the 13 candidate mutants (BmOr-1: Y170, D226, D299, E325, E356, D367, E375, D378; BmOrco: E171, E329, D343, E422, Y464) in combination with a paired wild type Or or Orco subunit.

**Figure 2:**
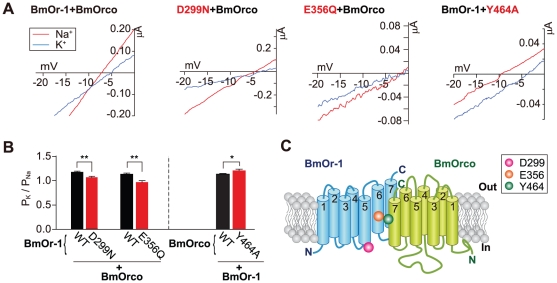
Mutations in BmOr-1 and BmOrco that affected ion selectivity. (**A**) Representative current-voltage (I–V) curves of oocytes expressing WT or mutant Or or BmOrco. Red and blue traces represent I–V curves obtained with Na^+^ and K^+^ solutions, respectively. (**B**) Summary of ion permeability ratios of the two BmOr-1 mutants (left) and the BmOrco mutants (right) that had a significant effect on ion selectivity (unpaired Student's t-test, mutant vs. WT **p<0.01; *p<0.05). Data on remaining mutants can be found in **Figure S2C**. Each bar represents mean ± S.E.M., n = 5–8. Bombykol was applied at the concentration of 1 µM to each oocyte. (**C**) Schematic showing the two mutations in BmOr-1 and one mutation in BmOrco that affected ion selectivity **(see also Figure S2C,S3B)**.

The P_K_/P_Na_ of two BmOr-1 mutants (D299N, E356Q) was slightly decreased compared with that of wild type (**Figure 2A,B**). The same decrease in P_K_/P_Na_ compared to wild type was found for the homologous BmOr-3 E365Q mutation (**Figure S3B**). In contrast, the P_K_/P_Na_ of BmOrco Y464A was slightly increased compared to wild type (**Figure 2A,B**). The other 10 candidate mutants did not show altered P_K_/P_Na_ (BmOr-1: Y170A, D226N, E325Q, D367N, E375N, D378N; BmOrco: E171Q, E329Q, D343N, E422Q) (**Figure S2C**). Therefore only a small number of the mutations examined here—BmOr-1 D299, BmOr-1 E356, and BmOrco Y464—altered the ion selectivity of the BmOr-1-BmOrco complex (**Figure 2C**), although the remaining 10 residues may be involved in selectivity of ions other than K^+^.

### Analysis of accessibility of methane thiosulfonate reagents to BmOr-BmOrco Cys mutants

We next used the Cys-modifying reagent, 2-(trimethylammonium)ethyl methanethiosulfonate, bromide (MTSET), to examine the accessibility of Cys mutants of BmOr-1 D299, BmOr-1 E356, and BmOrco Y464 to this modifying reagent. MTSET is a reagent that covalently modifies Cys residues that can be used to probe the functional properties of an ion channel [39]. If the targeted Cys is located in the channel pore, MTSET modification can affect ion permeability [39].

**Figure 3:**
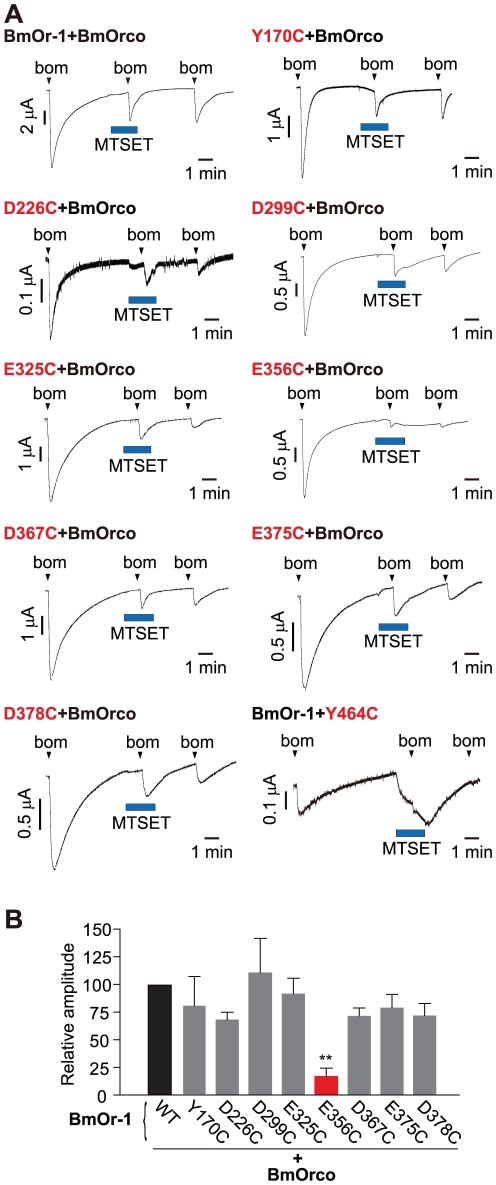
Effect of MTSET on ionic permeability of WT and Cys mutant BmOr-1-BmOrco complexes. (**A**) Representative current traces of oocytes expressing WT or mutant BmOr-1 or BmOrco. Bombykol (1 µM) and MTSET (2.5 mM) were applied at the time indicated by arrowheads and blue squares, respectively. (**B**) Summary of effects of MTSET on the bombykol response of WT (black bar) and mutant BmOr-1 (gray bars). The mutant with a significant effect on ion permeability is colored in red (unpaired Student's t-test, mutant vs. WT **p<0.01). Data are shown as mean ± S.E.M., n = 5 **(see also Figure S4)**.

We first determined the sensitivity of wild type BmOr-1-BmOrco to MTSET. The responsiveness of oocytes expressing wild type BmOr-1-BmOrco to bombykol was reduced 46% by MTSET (**Figure 3A**), perhaps due to modification of native Cys residues (data not shown). We next compared the effect of MTSET on BmOr-1 D299C, BmOr-1 E356C, and 6 other BmOr-1 mutants (Y170C, D226C, E325C, E367C, E375C, D378C) in combination with wild type BmOrco (**Figure 3A,B**). With the exception of BmOr-1 E356C, all other BmOr-1 Cys mutants showed a wild type response magnitude to bombykol in the presence of MTSET (**Figure 3A,B**). In contrast, the response of BmOr-1 E356C was strongly and irreversibly inhibited by MTSET application (**Figure 3A,B**). To exclude the possibility that the BmOr-1 E356C mutation affects ion permeation properties indirectly by a global change in protein structure, we determined the dose-response curve of BmOr-1 E356C to wild type BmOr-1. The EC_50_ value of the BmOr-1 E356C mutant was indistinguishable from wild type (**Figure S4A**), suggesting that the BmOr-1 E356C mutation does not affect ligand binding.

We next examined the effect of MTSET modification on the BmOrco Y464C mutant. In contrast to oocytes expressing wild type BmOr-1-BmOrco, which showed no activation by MTSET (**Figure 3A**), oocytes expressing wild type BmOr-1 with BmOrco Y464C showed a rapid inward current induced by MTSET (**Figure 3A**). A similar response to cysteine-modifying reagents has been observed for cyclic nucleotide-gated channels when Cys mutant residues were located in the ion channel pore domain [40]. The EC_50_ value of the BmOrco Y464C mutant was indistinguishable from wild type (**Figure S4B**). Taken together, these results show that BmOr-1 E356 in transmembrane domain (TM) 6 and BmOrco Y464 in TM7 are the only residues examined in this study that are sensitive to the MTSET Cys modifying reagent.

### Analysis of corresponding TM7 Tyr mutation in *Drosophila* Orco

**Figure 4:**
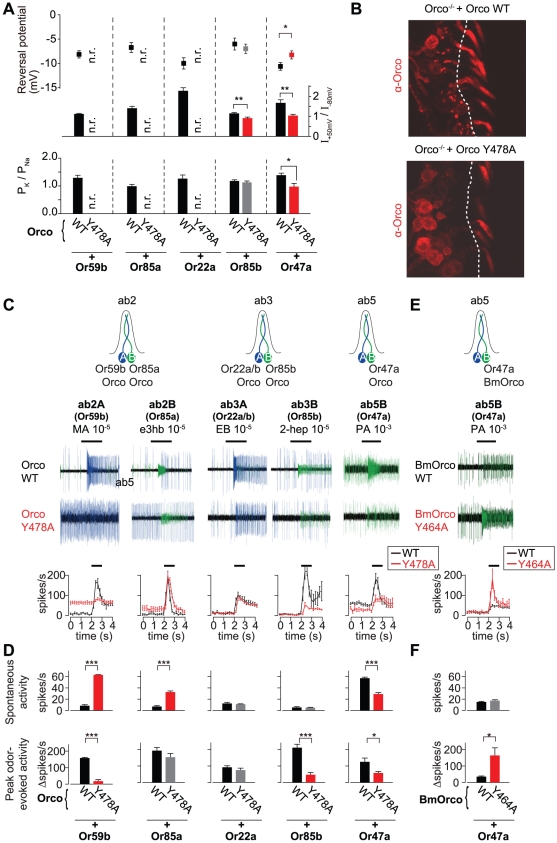
Effect of the *Drosophila* Orco Y478A mutation on Or-Orco function in heterologous cells and in *Drosophila* olfactory neurons. (**A**) Reversal potential (top), rectification index (middle), and permeability ratio (bottom) of oocytes expressing WT *Drosophila* Orco (black bars and symbols) or *Drosophila* Orco Y478A (red bars and symbols) along with WT ligand-selective Ors. Unpaired Student's t-test, mutant vs. WT, *p<0.05, **p<0.01. n.r. = no response. Data are shown as mean ± S.E.M., n = 8–10. (**B**) Anti-Orco antibody staining of WT *Drosophila* Orco (left) and *Drosophila* Orco Y478A expressed in *Orco^−/−^* animals. (**C**) (Top) Schematic of antennal sensilla showing the sensilla type and associated ligand-selective Ors. (Middle) Representative single-sensillum traces of ab2A, ab2B, ab3A, ab3B, ab5B neurons expressing WT *Drosophila* Orco or *Drosophila* Orco Y478A to methyl acetate, ethyl-3-hydroxybutyrate, ethyl butyrate, 2-heptanone, and pentyl acetate, respectively. Blue traces represent ab2A and ab3A neuron; green traces represent ab2B, ab3B, and ab5A and B neurons. (Bottom) Peristimulus time histograms of recordings from WT (black) and mutant (red). Data are presented as mean ±S.E.M., n = 3–7, and raw spikes/sec are plotted. (**D**) Summary of effects of WT *Drosophila* Orco (black bars) and *Drosophila* Orco Y478A (red bars) on spontaneous activity (top; raw spikes/sec) and odor-evoked activity. (bottom; corrected to subtract spontaneous activity). Unpaired Student's t-test, mutant vs. WT, *p<0.05, **p<0.01, ***p<0.001. Data are shown as mean ±S.E.M., n = 3–7. (**E**) (Top) Schematic of ab5 sensilla expressing WT BmOrco or BmOrco Y464A. (Middle) Representative single-sensillum traces of ab5 neurons expressing WT BmOrco or BmOrco Y464A to pentyl acetate. Green traces represent ab5A and ab5B neurons. (Bottom) Peristimulus time histograms of recordings from WT (black) and mutant (red). Data are presented as mean ±S.E.M., n = 4–5, and raw spikes/sec are plotted. (**F**) Summary of effects of WT BmOrco (black bars) and BmOrco Y464A (red bars) on spontaneous activity (top; raw spikes/sec) and odor-evoked activity. (bottom; corrected to subtract spontaneous activity). Data are shown as mean ± S.E.M., n = 4–5. Unpaired Student's t-test, mutant vs. WT, *p<0.05, **p<0.01, ***p<0.001.

To ask if the function of BmOrco Y464 is conserved in other insects, we mutated the corresponding Y478 residue in *Drosophila* Orco [19] (**Figure S1B**). We expressed *Drosophila* Orco Y478A along with one of five wild type *Drosophila* Ors (Or22a, Or47a, Or59b, Or85a, or Or85b) in oocytes, and measured rectification index, reversal potential, and ion selectivity of these channels gated by their cognate ligands (ethyl butyrate, pentyl acetate, methyl acetate, ethyl-3-hydroxybutyrate, or 2-heptanone, respectively) [5] (**Figure 4A**). *Drosophila* Orco Y478A combined with Or59b, Or85a, and Or22a did not respond to odor ligands, precluding further functional analysis (**Figure 4A**). Or85b expressed with *Drosophila* Orco Y478A showed a decreased rectification index but no effect on reversal potential or ion selectivity when compared to Or85b expressed with wild type *Drosophila* Orco (**Figure 4A**). Or47a expressed with *Drosophila* Orco Y478A showed a decreased rectification index, an increased reversal potential, and decreased P_K_/P_Na_ compared to Or47a expressed with wild type *Drosophila* Orco (**Figure 4A**). This suggests that mutating the TM7 Y478 residue in *Drosophila* Orco causes biophysical phenotypes that depend on the particular Or-Orco complex being studied.

### Analysis of *Drosophila* Orco Y478A and BmOrco Y464A mutants in *Drosophila* sensory neurons

Heterologous expression of Or-Orco in *Xenopus* oocytes allowed us to measure the effect of single amino acid mutations on several biophysical properties of the complex, such as rectification index, reversal potential, and ion selectivity. To ask if mutation of the conserved Y478 residue in *Drosophila* Orco (and the homologous BmOrco Y464A mutation) affected the function of the Or-Orco complex *in vivo*, we turned to extracellular recordings of spontaneous and odor-evoked action potentials of *Drosophila* OSNs expressing wild type Orco or Orco Y478A. This Orco mutation might impact spontaneous or odor-evoked action potentials, or both, *in vivo*.

To examine the effect of the Orco Y478A mutation *in vivo*, we generated transgenic flies that expressed wild type or mutant *Drosophila* Orco in all Orco-expressing OSNs. This was achieved using the Gal4/UAS system [41] to express wild type and Orco Y478A under the control of Orco-Gal4 in *Orco* null mutant flies [19]. For control experiments, we expressed wild type Orco using Gal4/UAS in the same Orco mutant genetic background. We first confirmed that *Drosophila* Orco Y478A is expressed at comparable levels to wild type *Drosophila* Orco and is appropriately trafficked to OSN dendrites in the antenna (**Figure 4B**).

We next recorded from ab2A, ab2B, ab3A, ab3B and ab5B OSNs, which express endogenous wild type Or59b, Or85a, Or22a, Or85b and Or47a, respectively [3], [4] (**Figure 4C,D**). Similar to what we observed for Orco Y478A in oocytes, we found a diversity of functional effects that depended on the specific Or-Orco complex. The ab2A Or59b neuron expressing Orco Y478A showed a large increase in spontaneous activity and a strong decrease in responses evoked by methyl acetate, an odorant that elicits an excitatory response in wild type ab2A neurons [5] (**Figure 4C,D**). In contrast, in the ab2B neuron expressing Or85a, the Orco Y478A mutation caused a change in spontaneous activity with no effect on odor-evoked activity (**Figure 4C,D**). The reverse phenotype was found for Orco Y478A expressed in ab3B OSNs expressing Or85b, such that the Or85b-Orco Y478A receptor had wild type spontaneous activity but a strong decrease in odor-evoked activity (**Figure 4C,D**). The ab5B neuron expressing Or47a showed a decrease in both spontaneous and odor-evoked activity (**Figure 4C,D**). Finally, there was no effect of the Orco Y478A mutation on spontaneous or evoked responses in the ab3A neuron expressing Or22a (**Figure 4C,D**).

To ask if this effect of the Orco TM7 Tyr residue on the *in vivo* function of *Drosophila* Orco was conserved, we expressed wild type BmOrco or BmOrco Y464A in an *Orco* null mutant background (**Figure 4E,F**). Wild type BmOrco in combination with native *Drosophila* Or59b, Or85a, Or22a, Or85b, and Or47a in ab2A, ab2B, ab3A, ab3B, and ab5B OSNs, respectively, showed normal responses to the cognate odor ligand of each OSN (data not shown), suggesting that silkmoth BmOrco can function with endogenous *Drosophila* Ors as previously shown for Orco from several other insect species [42]. We then turned to the functional analysis of BmOrco Y464A. This mutant combined with Or59b, Or85a, Or22a, and Or85b did not respond to odor ligands, precluding further functional analysis (data not shown). However, BmOrco Y464A expressed with Or47a showed normal spontaneous activity but a strong increase in odor-evoked activity (**Figure 4E,F**).

Taken together, these results suggest that the TM7 Tyr residue in Orco (Y478 in *Drosophila* and Y464 in *Bombyx*) contributes to the function of the insect Or-Orco complex *in vivo*, although the function of this Tyr residue may not be identical between DmOrco and BmOrco. Because the effect of the Orco Y478A mutation differed depending on the Or-Orco complex, we suggest first, that each insect Or-Orco complex possesses different functional properties and second, that both the ligand selective Or subunit and the Orco co-receptor subunit contributes to channel activity.

## Discussion

In this study, we carried out comprehensive site-directed mutagenesis of all conserved Glu, Asp, and Tyr residues in the silkmoth bombykol receptor to probe the structure-function relationships of the Or-Orco complex. 13 of the 83 residues caused functional alterations in odor-evoked cation channel activity. Furthermore, three of the 13 residues showed altered ion selectivity. Two of the residues were located in transmembrane domain (TM) TM5 and TM6 in a ligand-selective Or and a third was in TM7 in Orco. These three residues may contribute to ion permeability of the receptor complex, although our data cannot resolve whether these three residues are part of an ion-conducting pore or merely influence the function of a pore residing elsewhere in the protein complex.

Pore domains of cation channels are typically formed by the assembly of multiple subunits [37]. Insect Or-Orco functions as a heteromultimer [12], [22], [23], but the stoichiometry and subunit composition of the complex are unknown. Further, it is unclear whether the ion-conducting pore structure of Or-Orco complex is formed by Orco alone or whether both ligand-selective Ors and Orco contribute to the pore. There is evidence that the Orco subunit alone can form an ion channel [29], [33], but there is also suggestive evidence that the ligand-selective Or contributes to the ionic properties of the Or-Orco complex. First, sensitivity of Or-Orco to ruthenium red varies with Or subunit composition [34], [36]. Second, the reversal potential of *Anopheles* Orco alone is slightly larger than that of the *Anopheles* Or10-Orco complex, indicating that ion selectivity is modulated by the ligand-selective Or subunit [33]. In this study, we provide confirmatory evidence that both subunits contribute to ionic permeability of the insect Or-Orco complex.

Our work suggests that the Or-Orco complex has two important characteristics. First, the biophysical properties of the channel vary according to subunit composition, even with highly similar proteins such as BmOr-1-Orco and BmOr-3-Orco. Second, because ligand-selective Or sequences within and between insect species are extremely divergent, the primary amino acid sequence of the ion-conducting pore is likely to differ according to the subunit composition of the Or-Orco complex. This is consistent with our model that the ion pore of the insect Or requires the participation of both Or and Orco subunits. Neither Orco nor any ligand-selective Or has been found to have any homology to known ion-conducting pore domains in other ion channels. This suggests that insect Ors will define a completely novel structural domain for ion selectivity and permeability not found in other ion channels. Further experiments will be needed to understand the precise pore structure of the Or-Orco complex. A comprehensive substituted cysteine accessibility study could reveal amino acid residues that reside in the pore lumen. High-resolution x-ray structural analysis of open and closed channel states would inform the stoichiometry of the complex and the mechanisms underlying ion conduction of this unusual family of ion channels.

## Materials and Methods

### Odor ligands and protein modification reagents for heterologous expression analysis

Bombykol and bombykal were synthesized as previously reported [38] and solutions of these two compounds were prepared in dimethylsulfoxide (DMSO). Other odorants were purchased from Tokyo Kasei (Tokyo, Japan) and were directly diluted into control bath solution (88 mM NaCl, 1 mM KCl, 0.3 mM Ca(NO_3_)_2_, 0.4 mM CaCl_2_, 0.8 mM MgSO_4_, 2.4 mM NaHCO_3_, 15 mM HEPES, pH 7.6) at a final working concentration of 1 mM. The Chemical Abstracts Service (C.A.S.) number of odorants used in this study are: methyl acetate (79-20-9), ethyl-3-hydroxybutyrate (5405-41-4), ethyl butyrate (105-54-4), 2-heptanone(110-43-0), pentyl acetate (628-63-7). MTSET [2-(trimethylammonium)ethyl methanethiosulfonate, bromide] was purchased from Anatrace Inc. (Maumee, OH, USA). Prior to each experiment, dry MTSET powder was dissolved in ice-chilled control bath solution at a stock concentration of 250 mM. This stock solution was diluted to 2.5 mM just before application to each oocyte because MTSET decomposes in solution quickly. BMS [bis(2-mercaptoethyl)sulfone] was purchased from Santa Cruz Biotechnology Inc. (Santa Clara, CA, USA) and was diluted directly into control bath solution to a final working concentration of 5 mM.

### Site-directed mutagenesis

Point mutations were introduced into insect Ors (BmOr-1, BmOr-3, BmOrco, *Drosophila* Orco) by PCR using a reaction mixture containing KOD-Plus-buffer (Toyobo, Tokyo, Japan), 0.2 mM of each dNTP, 1 mM of MgSO_4_, 20 ng of cDNA, 0.5–1 µM oligonucleotide primers, and 1 U of KOD–Plus (Toyobo, Tokyo, Japan). All of the single point mutation products were digested by restriction enzymes (BmOr-1: *Bgl*II/*Xho*I; BmOr-3, BmOrco, *Drosophila* Orco: *Eco*RI/*Xho*I), and the resulting fragments were inserted into the multicloning site of the modified pSPUTK vector, which was utilized to synthesize cRNAs for *Xenopus* oocyte injection [43]. All mutations were confirmed by DNA sequencing using a BigDye Terminator v3.1 Cycle Sequencing kit (Applied Biosystems, Foster City, CA).

### Gene expression in *Xenopus laevis* oocytes and two-voltage clamp recording

Stage V to VII oocytes were treated with 2 mg/ml of collagenase B (Roche Diagnostics, Tokyo, Japan) in Ca^2+^-free saline solution (82.5 mM NaCl, 2 mM KCl, 1 mM MgCl_2_, and 5 mM HEPES, pH 7.5) for 1 to 2 h at room temperature. cRNA was synthesized from linearized modified pSPUTK vector. Oocytes were microinjected with 25 ng of BmOr-1 or BmOr-3 cRNA and 25 ng of cRNA BmOrco or *Drosophila* Orco. Injected oocytes were incubated for 3–4 days at 18°C in bath solution supplemented with 10 µg/ml of penicillin and streptomycin.

Whole-cell currents were recorded using the two-electrode voltage-clamp technique as previously described [12]. Intracellular glass electrodes were filled with 3 M KCl. Signals were amplified with an OC-725C amplifier (Warner Instruments, Hamden, CT, USA), low-pass filtered at 50 Hz and digitized at 1 kHz. The control bath solution contained 115 mM NaCl, 2.5 mM KCl, 1.8 mM BaCl_2_, and 10 mM HEPES, titrated to pH 7.2 with NaOH.

The rectification index was calculated as the ratio of current amplitude recorded at +50 mV versus amplitude at −80 mV. In ion substitution experiments, the following solutions were used: 115 mM XCl, 10 mM HEPES, titrated to pH 7.2 with XOH (X = Na^+^ or K^+^). To calculate the permeability ratio, the following extended form of the Goldman-Hodgkin-Katz flux equation was used: P_K_/P_Na_ = [Na]_o_/[K]_o_·exp(ΔE_rev_·F/RT). In the measurement of E_rev_, the junction potential was corrected. In the experiment using MTSET reagent, each oocyte was pretreated with MTSET (2.5 mM) for 60 seconds before the application of bombykol. Ligands were delivered through the superfusing bath solution via a silicon tube connected to a computer-driven solenoid valve. Data acquisition and analysis were carried out with Digidata1322A (Axon instruments, Foster city, CA, USA) and pCLAMP software (Axon instruments, Foster city, CA, USA).

### Fly strains and transgenic constructs

*Drosophila melanogaster* stocks were maintained on conventional cornmeal-agar-molasses medium under a 12 hour light∶12 hour dark cycle at 25°C. Transgenic animals were generated in the *w^1118^* genetic background (Genetic Services Inc., Cambridge, MA, USA) using the phiC31-based integration system [44] targeted to the attP2 docking site on chromosome II [45]. UAS-Orco [22] and Orco-Gal4 [19] transgenes were randomly integrated via conventional P-element vectors.

Transgenes were crossed into a transheterozygous *Orco^1^*/*Orco^2^* mutant background [19] using standard fly genetics. The Gal4/UAS system [41] was used to cross UAS-OrcoY478A and UAS-BmOrcoY464A together with Orco-Gal4 into the *Orco* mutant background.

### Single sensillum electrophysiology and odorants

Female transgenic flies were recorded between 5 and 7 days after adult eclosion. Single sensillum recordings were performed as described [46], [47].

Odorants were obtained from Sigma-Aldrich (St. Louis, MO, USA) at high purity and diluted (v/v) in paraffin oil as indicated. Chemical Abstracts Service (CAS) numbers: paraffin oil (8012-95-1); 2-heptanone (110-43-0); methyl acetate (79-20-9); ethyl-3-hydroxybutyrate (5405-41-4); ethyl butyrate (105-54-4); pentyl acetate (628-63-7).

30 µl of the desired odor dilution was pipetted onto a filter paper strip (3×50 mm), which was then carefully inserted into a glass Pasteur pipette. Prior to any recordings, charcoal-filtered air was forced through the pipette for 1–3 sec to remove dead space in the odor delivery system. For actual recordings, charcoal-filtered air was continuously applied to the insect antenna, with odor delivered through the pipette to the fly antennae for 1 sec. Each pipette was used at most three times and no more than three sensilla were tested per animal. Sensilla types were identified by size, location on the antenna, and responsiveness to known preferred odorants [5].

Data were collected using Autospike software (Syntech, Kirchzarten, Germany) and analyzed by custom spike sorting algorithms [46]. Spikes from the ab5A and B neurons were not sorted because of the similarity in spike amplitudes. The data were analyzed by calculating the number of spikes/sec in 200 msec bins. The peak odor-evoked activity was calculated by subtracting the average spontaneous activity (expressed in spikes/sec) during the two seconds before odor application from peak activity during odor delivery. This value is referred to as Δspikes/sec. The onset of odor-evoked responses varied due to slight variations in the position of the odor delivery system relative to the sensillum being recorded. To correct for this, we calibrated the inferred odor onset for each sensillum recorded based on excitatory responses for each sensillum elicited by control stimuli (ab2: 10^−5^ methyl acetate; ab3: 10^−5^ 2-heptanone; ab5: 10^−2^ geranyl acetate).

### Histology

Antibody staining was performed on 14 µm frozen antennal sections of transgenic 5–7 day old *Drosophila* animals according to standard protocols [19]. Orco staining was performed using an antibody that recognizes the 2^nd^ extracellular loop of *Drosophila* Orco (dilution 1∶5000; [22]), revealed by secondary Cy3-conjugated goat α-rabbit IgG antibodies (Jackson ImmunoResearch, West Grove, PA, USA, dilution 1∶200). Visualization was performed using a Zeiss LSM510 confocal microscope (Carl Zeiss Jena GmbH, Jena, Germany).

### Statistics

Statistical analysis in Figure 1, 2, 3, 4A, S2, S3, S4 was performed in Prism software (GraphPad Software, Inc., La Jolla, CA, USA) using an unpaired Student's t-test. Comparisons in Figure 4D, 4F were performed in Excel using an unpaired Student's t-test.

## Supporting Information

Figure S1 **Amino acid alignment of insect pheromone receptors and Orco from various insect species.** (**A**–**B**) Amino acid sequences of various insect pheromone receptors (A) and Orco in various insect species (B) were aligned manually. Negatively-charged residues (E, D) and Y are indicated in red and green, respectively. Putative transmembrane domains, predicted by the PHDhtm algorithm ([48]), are marked with gray squares. Residues conserved in all aligned proteins are indicated with open squares. Arrowheads indicate the residues targeted for site-directed mutagenesis. NCBI accession numbers for (A) are as follows: BmOr-1: NM_001043410, BmOr-3: NM_001043460, OscaOR1: AB467320, OscaOR3: AB508293, OscaOR4: AB508294, HR13: AJ748328, PxOR1: AB263116, DiOR1: AB263113, EpOR1: EU791886. NCBI accession numbers for (B) are as follows: *Bombyx. mori*: NM_001043595, *Ostrinia scapulalis*: AB467318, *Heliothis virescens*: AJ487477, *Helicoverpa zea*: AY843204, *Drosophila melanogaster*: NM_079511, *Ceratitis. capitata*: AY843206 *Anopheles gambiae*: AY363725, *Apis mellifera*: NM_001134943.
(EPS)

Figure S2 **Mutations in BmOr-1 and BmOrco with no effect on rectification index, reversal potential and ion selectivity.** (**A**) Schematic drawing of the location of mutated amino acids in BmOr-1 and BmOrco. Transmembrane domains were predicted using the PHDhtm algorithm. (**B**) Rectification index and reversal potential of oocytes expressing mutant BmOr-1 and WT BmOrco (left) and WT BmOr-1 and mutant BmOrco (right). Black and gray bars and symbols represent the rectification index of WT and mutants, respectively. Those mutants with significantly different rectification index from WT are indicated as red bars (unpaired Student's t-test, *p<0.05 mutant vs WT). Data are shown as mean ± S.E.M, n = 8–10. Bombykol was applied at the concentration of 1 µM to each oocyte. (**C**) Summary of ion permeability ratios of BmOr-1 (left) and BmOrco mutants (right) with no significant effect on ion selectivity (unpaired Student's t-test, mutant vs. WT p>0.05). Data are plotted as mean ± S.E.M., n = 5. Black bars indicate WT and gray bars indicate mutants. Bombykol was applied at the concentration of 1 µM to each oocyte.
(EPS)

Figure S3 **Effects of mutations of candidate amino acids in BmOr-3.** (**A–B**) Reversal potential (A, top), rectification index (A, bottom), and permeability ratio (B) of oocytes expressing WT (black bar) or mutant BmOr-3 (red bars) with WT BmOrco. Unpaired Student's t-test, *p<0.05, ***p<0.001 mutant vs WT. Data are shown as mean ± S.E.M., n = 10. n.r. = no response.
(EPS)

Figure S4 **Dose-response curves of BmOr-1 E356C and BmOrco Y464C.** (**A–B**) Dose response curves of oocytes expressing BmOr-1 E356C with WT BmOrco (A), WT BmOr-1 with BmOrco Y464C (B) compared to WT BmOr-1-BmOrco. Bombykol was sequentially applied to the same oocytes at the indicated concentrations. Each point represents the mean current value ± S.E.M. from 3–4 individual oocytes and is not significantly different between WT and mutants (n = 3, p>0.05, unpaired Student's t-test). EC50 values are as follows: BmOr-1+BmOrco: 0.19 µM; E356C+BmOrco: 0.30 µM, BmOr-1+Y464C: 0.16 µM.
(EPS)
